# Mentorship: Remembering My Mentor Guy Caldwell and His Mentor Michael Hoke

**DOI:** 10.31486/toj.22.5023

**Published:** 2022

**Authors:** M. Mark Hoffer

**Affiliations:** Emeritus Professor of Orthopaedic Surgery, University of California–Irvine, and Lowman Professor of Orthopaedic Hospital of Los Angeles

## TO THE EDITOR

I had the great fortune to spend my 4 years of orthopedics training (1964-1968) at the Ochsner Clinic. My chiefs, Dr Morris and then Dr Dunn, were excellent surgeons and teachers. They were extremely kind to house staff and patients. I had the unique added privilege to spend part of my first year as Dr Guy Caldwell's last resident. Dr Caldwell had retired but came back to follow the patients of Dr Mary Sherman who had unexpectedly passed away. I had been assigned as her resident, and Dr Caldwell and I spent a great deal of time reviewing her cases and organizing surgical procedures with the attending surgeons. We found time to talk about the history of children's orthopedics and the influence of Dr Caldwell's mentor Dr Michael Hoke.

Michael Hoke (1874-1944) was the son of a southern Civil War general and the nephew of Mayor Van Wyck of New York City. He was born and raised in North Carolina, graduated from medical school in Virginia, did a surgical residency at Johns Hopkins, and completed a year of orthopedics training in Boston, Massachusetts. He established a private practice and consulted in Atlanta, Georgia. In 1915, Dr Hoke became the medical director of a new Atlanta hospital for physically disabled children. That hospital developed a Masonic sponsorship and became one of the first of a few Scottish Rite institutions. At the 1920 Shriners Convention in Portland, Oregon, Dr Hoke presented an illustrated talk describing the challenges and work done at his Atlanta institution. The entire group supported a resolution to develop and finance a series of Shriners hospitals for these children. Shriners Hospitals for Children subsequently developed facilities throughout the United States. In 1931, Dr Hoke accepted President Franklin Roosevelt's invitation to become the first medical director at the Roosevelt polio rehabilitation hospital in Warm Springs, Georgia.

Guy Caldwell (1891-1981) was a native of Mississippi who obtained his medical degree with an internship and residency at Columbia University Presbyterian Hospital. When he finished his training in 1915, he volunteered as a military surgeon and left for France. In 1917, he became an American Army surgeon and was given added supervisory responsibilities because of his war experience. After the war, he began a practice in Atlanta, worked at Emory University, and learned from his mentor, Dr Hoke. In 1922, Dr Hoke arranged for Dr Caldwell to become medical director of the first of the Shriners hospitals, located in Shreveport, Louisiana. For 16 years, Dr Caldwell worked at that Shriners hospital in addition to maintaining a practice and teaching at the local charity hospital. In 1938, he joined Tulane Medical School as chair of the new separate orthopedics department. His residency became one of the most sought after in the country. His extensive trauma experience during the war and pediatric orthopedics experience with the early Shriners hospital made him a mentor for students, residents, and colleagues. During World War II, he was asked to act as advisor to the Surgeon General of the United States Army. His administrative talents helped establish the Ochsner Clinic, which is now a worldwide-respected institution.

After a new attending surgeon was hired to follow Dr Sherman's patients, Dr Caldwell and I still found time to talk of children's orthopedics. Before I moved to California, Dr Caldwell referred me to his old friend Dr Charles Lowman, chief surgeon at the Orthopaedic Hospital in Los Angeles. I spent more than half a century as a children's orthopedic surgeon, and I credit my time at Ochsner Clinic and mentorship by Dr Caldwell for a long and happy career.

